# Noninvasive Imaging of Cardiac Electrophysiology

**Published:** 2007-08-01

**Authors:** Thomas Berger, Florian Hintringer, Gerald Fischer

**Affiliations:** 1 Dep. of Internal Medicine, Div. of Cardiology, Innsbruck Medical University, Innsbruck, Austria; 2 Institute for Biomedical Engineering, Medical Informatics and Technology (UMIT), Hall i. Tirol, Austria

**Keywords:** noninvasive imaging, cardiac electrophysiology

## Abstract

Noninvasive imaging of cardiac electrophysiology is still a major goal despite all recent technical innovations. This review gives an overview about the historical background, recent developments and possible future applications of noninvasive imaging of cardiac electrophysiology.

## Standard Electrocardiogram (ECG)

The basic research according the standard 12-lead electrocardiogram (ECG) dates from the Dutch physician Einthoven, who used a string galvanometer to record myocardial currents at about the year 1900. The so called "Einthoven" leads depict potential differences between the right and the left arm (lead I), the right arm and the left leg (lead II), and the left arm and the left leg (lead III). Later on, Goldberger introduced three more leads providing three additional projections of the electrical heart vector (aVL, aVR and aVF). The Goldberger and Einthoven leads provide bipolar signals, as electrical potential differences of two distinct sites are obtained. Frank N. Wilson and coworkers developed a vacuum tube amplifier which allowed recordings of potential variations at single leads placed on the anterolateral chest. Therefore, Wilson introduced an "indifferent electrode", the "Wilson central terminal (WCT)". Contrary to the Einthoven and Goldberger leads, the signals measured between the Wilson leads V1-6 and the indifferent WCT are unipolar signals. Due to the anatomical localization on the chest, the Wilson leads provide higher signal amplitudes and give some additional information about spatial differences of cardiac electrophysiology. So far, the 12-lead ECG remains the primary clinical tool for noninvasive assessment of cardiac electrophysiology and there have been only little clinical developments, except for stress testing and Holter ECG, over the last decades.

## High Resolution ECG- Body Surface Potential Mapping

To increase spatial resolution of the body surface electrocardiogram, different studies have tried to increase the sampling density by increasing the number of ECG leads [1]. For visualization of the increased data of the high-density ECG, body surface potential maps have been introduced. Body surface mapping data are visualized as plots of potential maps for each time step [2]. The temporal evolution of potential differences on the body surface contains information about the underlying cardiac electrophysiology. Although, routine use of these high-density body-surface maps is not practicable due to missing standards of the mapping arrays [3] - and the problem of comparability of the different map patterns (few databases for different case-dependent map patterns). Moreover, the number of leads needed for optimal spatial resolution as well as the optimal electrode location remain still point of discussion [4]. Another limitation of clinical usability of high density lead arrays is the time needed for application of the lead array. Though, the design of commercial electrode vests may overcome these problems. Other prerequisites for body surface mapping are appropriate software tools for processing the large amounts of data acquired by the high-density ECG lead arrays.

## Epicardial Activation Maps

A further progress in imaging of cardiac electrophysiology was the introduction of field computation techniques such as the finite element method. It has been demonstrated that a single patch of tissue, when  activated by pacing, results in a negative potential. Consecutively, a sharp drop of activation occurs in the resulting activation wave front. Immediately, the potential magnitude changes to positive or negative values in the order of some milivolts. This results in the occurrence of two potential maxima. The maxima mark the activated wavefront, and an imaginary line connecting the maxima is parallel to fiber orientation [5]. Although, this approach provides valuable insights into the basics of cardiac electrophysiology and ECG mapping, this method did not reach widespread usage [1,6].

## Integral Maps

Integral maps are computed by the integral calculus of the potential in a defined time interval and its interpolation to the torso surface. QRS integrals represent the activation sequence (upstroke in the action potential), ST-T integral reflect the repolarization sequence (downstroke in the action potential), and QRST integrals relate to differences in action potential integrals, independent of the activation sequence.

The QT-dispersion is a widely used parameter which reflects the heterogeneity of repolarization. Here, the QT time interval in each lead is assessed and a measure for dispersion of repolarization (longest - shortest QT interval) is assessed. One major limitation of QT dispersion is the poorly defined end of the T-wave, especially in leads with small T-wave amplitudes.

In the last years, intracardiac maps have gained special importance in clinical use for e.g. electrophysiological studies. Therefore, intracardiac maps are obtained with intracardiac catheters with integrated magnetic sensors (e.g. CARTO™), which allows recording of endocardial signals as well as the corresponding anatomical location of the catheter tip. This method allows electroanatomic imaging of activation time maps with sufficient spatiotemporal resolution using color-coded plots.

## Noninvasive Imaging of Cardiac Electrophysiology (NICE)

**Figure 1:**
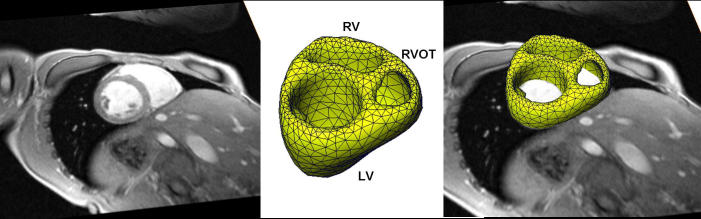
The left panel shows a MRI cardiac short axis scan (slice thickness 6mm). The middle panel shows a triangulated and remeshed surface model of individual cardiac anatomy (LV=left ventricle; RV=right ventricle; RVOT=right ventricular outflow tract). The right panel shows the fusion of MRI data and the individual cardiac model. (Figure modified from Berger T et al,  Single-beat noninvasive imaging of cardiac electrophysiology of ventricular pre-excitation. J Am Coll Cardiol 2006; 48: 2045-52. with permission. Copyright Elsevier, American College of Cardiology, 2006)

**Figure 2:**
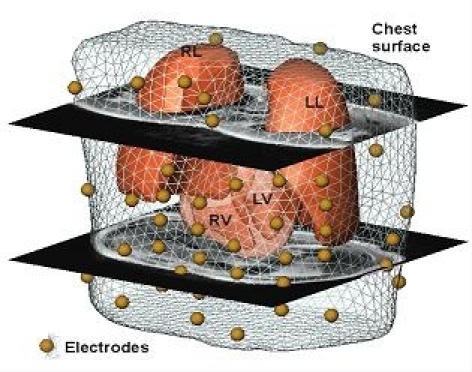
Localization of the 65-leads on the chest surface as used for the NICE approach. (Figure modified from Berger T et al,  Single-beat noninvasive imaging of cardiac electrophysiology of ventricular pre-excitation. J Am Coll Cardiol 2006; 48: 2045-52. with permission. Copyright Elsevier, American College of Cardiology, 2006)

**Figure 3:**
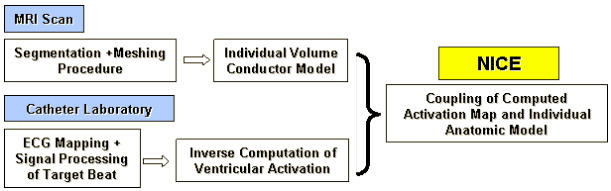
Workflow of NICE

This novel method uses an ill-posed mathematical theorem for an inverse projection of functional information contained in the body surface ECG to the epicardial source. As NICE is a functional imaging method, patient's individual cardiac anatomy is assessed by magnetic resonance imaging or computer tomography. An individual computer model of cardiac anatomy is computed for each patient (Figure 1). Additionally, the location of each lead of the high density body surface ECG (65 leads) is digitized and projected on the skin surface in the computed model (Figure 2). Thus, this method provides a fusion of anatomical imaging with functional data by solving an inverse mathematical problem (Figure 3). Many different approaches have been tried to solve this inverse problem using different source formulations (potentials, current densities, and activation times) and different side constraints for stabilizing the method with respect to noise [7,8]. Importantly, the source formulation and regularization approach has a major impact on stability of the method. Two different approaches have yet been successfully applied to patients.

## Electrocardiographic Imaging (ECGI)

Electrocardiographic imaging tries to calculate and display the electrical activity from the epicardium from surface ECG signals using a boundary element (BEM) or finite element method (FEM) for computation of the epicardial electrical activation. When considering this inverse mathematical problem a regularization method (Tikhonov) is used to solve this ill-posed problem [9,10]. Computation of endocardial activation is yet not possible, as the electrical signal amplitude from the midwall and endocardium is much smaller. Initially, ECGI was developed and validated in animal studies using a rigid human-shaped torso tank of homogeneous conductivity with an animal heart in a fixed position. A first application in humans has recently been published [11].

## Activation Time Imaging

In contrast to electrocardiographic imaging, which can be applied to any time interval of the cardiac activation, activation time imaging (ATI) is limited to the time interval of atrial or ventricular depolarization (P-wave, QRS complex). Early studies used an uniform dipole layer model for describing the source which uses the assumption of isotropic conductivity (a propagating activation wave produces a step-like change in the electrical potential on the cardiac surface=epicardium). More recently a bidomain model has been applied. The biophysically familiar action potential enters the source term and therefore it can also be applied (in principle) to anisotropic models of the myocardium.

## Action Potential Imaging

**Figure 4:**
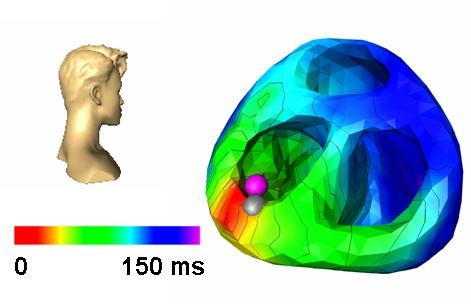
The areas of first onset of ventricular activation are indicated by red colour, the areas of latest activation are indicated by blue colour, as obtained by NICE. Here, preexcitation is indicated by early ventricular activation (red area) in a patient with a left posterolateral accessory pathway. The ablation points (grey markers) and the location of successful ablation (purple marker) indicating the ventricular insertion site of the accessory pathway, as obtained by CARTO™. Head icons indicate point of view. Isochrones are plotted in 20 ms intervals. (Figure modified from Berger T et al,  Single-beat noninvasive imaging of cardiac electrophysiology of ventricular pre-excitation. J Am Coll Cardiol 2006; 48: 2045-52. with permission. Copyright Elsevier, American College of Cardiology, 2006)

The strong constraint of a step-like activation function with a fixed amplitude and rise time limits the application of action potential imaging. A weaker but still strong limitation is that the action potential is restricted by a lower and upper bound (maximal amplitude) and monotonically increasing during activation [12]. By imposing the constraint of a monotonically increasing function, source components can be retrieved, which for electrocardiographic imaging are in the null space of the signal, which is of particular importance when extending ECGI to the entire heart surface, thus including the endocardium [13]. The approach has been successfully applied for imaging action potential in patients with overt Wolff-Parkinson-White syndrome and has been validated using catheter based electroanatomic imaging (CARTO™) [14]. As a first step, this study indicates the feasibility of noninvasive imaging of ventricular preexcitation (Figure 4). Further validation studies for different arrhythmogenic substrates are necessary in order to improve the proposed method and to establish NICE for clinical application in a catheter laboratory. NICE based imaging of ventricular activation is feasible with a spatial and temporal resolution which is competitive to invasive electroanatomic imaging techniques. Noninvasive imaging of cardiac electrophysiology may be used as a complementary noninvasive approach to localize the origin and help to identify and understand the underlying mechanisms of cardiac arrhythmias. Another future application may be the identification of responder to cardiac resynchronization therapy by noninvasive imaging of electroanatomical dyssynchrony. Further improvements in cardiac imaging (computer tomography, magnetic resonance imaging) may also help to further decrease procedural time. In conclusion, noninvasive imaging of cardiac electrophysiology may become a valuable diagnostic tool in cardiac electrophysiology as indicated by the first applications in humans.
